# Aryl Hydrocarbon Receptor Defect Attenuates Mitogen-Activated Signaling through Leucine-Rich Repeats and Immunoglobulin-like Domains 1 (LRIG1)-Dependent EGFR Degradation

**DOI:** 10.3390/ijms22189988

**Published:** 2021-09-15

**Authors:** Han-Lin Hsu, Hong-Kai Chen, Chi-Hao Tsai, Po-Lin Liao, Yen-Ju Chan, Yu-Cheng Lee, Chen-Chen Lee, Ching-Hao Li

**Affiliations:** 1 Division of Pulmonary Medicine, Department of Internal Medicine, Wan Fang Hospital, Taipei Medical University, Taipei 116, Taiwan; lisa11011117@gmail.com; 2 Pulmonary Research Center, Wan Fang Hospital, Taipei Medical University, Taipei 116, Taiwan; 3 School of Respiratory Therapy, College of Medicine, Taipei Medical University, Taipei 110, Taiwan; b117104067@tmu.edu.tw; 4 Department of Physiology, School of Medicine, College of Medicine, Taipei Medical University, Taipei 110, Taiwan; nkj31909@tmu.edu.tw; 5 Graduate Institute of Medical Sciences, College of Medicine, Taipei Medical University, Taipei 110, Taiwan; yclee0212@tmu.edu.tw; 6 Department of Ophthalmology, University of North Carolina School of Medicine, Chapel Hill, NC 27517, USA; d01447001@ntu.edu.tw; 7 Institute of Food Safety and Health Risk Assessment, School of Pharmaceutical Sciences, National Yang-Ming University, Taipei 112, Taiwan; plliao@ym.edu.tw; 8 Department of Microbiology and Immunology, School of Medicine, China Medicine University, Taichung 404, Taiwan; leechenchen1973@gmail.com

**Keywords:** aryl hydrocarbon receptor (AHR), epidermal growth factor receptor (EGFR), leucine-rich repeats and immunoglobulin-like domains 1 (LRIG1), cell proliferation, a disintegrin and metalloprotease 17 (ADAM17), chronic obstructive pulmonary disease (COPD)

## Abstract

Aryl hydrocarbon receptor (AHR) genomic pathway has been well-characterized in a number of respiratory diseases. In addition, the cytoplasmic AHR protein may act as an adaptor of E3 ubiquitin ligase. In this study, the physiological functions of AHR that regulate cell proliferation were explored using the CRISPR/Cas9 system. The doubling-time of the AHR-KO clones of A549 and BEAS-2B was observed to be prolonged. The attenuation of proliferation potential was strongly associated with either the induction of p27^Kip1^ or the impairment in mitogenic signal transduction driven by the epidermal growth factor (EGF) and EGF receptor (EGFR). We found that the leucine-rich repeats and immunoglobulin-like domains 1 (LRIG1), a repressor of EGFR, was induced in the absence of AHR in vitro and in vivo. The LRIG1 tends to degrade via a proteasome dependent manner by interacting with AHR in wild-type cells. Either LRIG1 or a disintegrin and metalloprotease 17 (ADAM17) were accumulated in AHR-defective cells, consequently accelerating the degradation of EGFR, and attenuating the response to mitogenic stimulation. We also affirmed low AHR but high LRIG1 levels in lung tissues of chronic obstructive pulmonary disease (COPD) patients. This might partially elucidate the sluggish tissue repairment and developing inflammation in COPD patients.

## 1. Introduction

The activities of epithelial cells respond to a subset of growth factors in their surrounding environment. The epidermal growth factor (EGF) and EGF receptor (EGFR, also called the ErbB-1/HER1) play pivotal roles that program the cellular machinery to proliferate, differentiate, and cause tissue development. EGFR belongs to the erbB receptor tyrosine kinase (RTK) family, and is ubiquitously expressed in epithelial, mesenchymal, and neuronal cells. Upon the binding of soluble EGF peptide to the extracellular domain, the EGFR preferentially dimerizes with their cognate receptor, the erbB2, which is constitutively active without any identifiable ligand binding. The dimerization of EGFR and erb2 is an essential step for the autophosphorylation of key tyrosine residues within the cytoplasmic portion of EGFR. This activates the tyrosine kinase activity and initiates intracellular signaling pathways involving mitogen-activated protein kinase (MEK)/mitogen-activated protein kinase (MAPK) pathway, phosphoinositide-3 kinase/protein kinase B (Akt) pathway, and Janus kinase (Jak)/signal transducer and activator of transcription (STAT) pathway. Aside from the EGF, a multitude of ligands (e.g., amphiregulin, transforming growth factor-alpha) have been reported to bind to EGFR, imparting a variety of biological responses in different organs [1,2]. The abnormalities of EGFR, including protein over-expression and aberrant activation (mainly due to mutations), usually enable an overgrowth signal. As a consequence, the uncontrolled proliferation, apoptosis resistance, sustained angiogenesis, improved cell migration, and epithelial-to-mesenchymal transition (EMT) are displayed in a wide variety of human blastoma [3], sarcoma [4], and carcinomas [5]. Actually, EGFR mutations are found in 40% of lung cancers [2], suggesting that EGFR might be identified as a selective target during cancer chemotherapy.

The aryl hydrocarbon receptor (AHR) is a nucleocytoplasmic shuttling protein. The nuclear import of AHR is triggered by ligand binding. Most synthetic ligands are environmental pollutants (e.g., dioxins and benzo[a]pyrene) that are powerful AHR activators. Ligand-dependent transactivation of AHR (the genomic pathway) has been extensively studied in the biotransformation of xenobiotics, carcinogenesis, and the pathogenesis of several disorders [6,7]. The lungs are vulnerable organs and are highly responsive to AHR ligands; the airway epithelium is the first structure exposed to inhaled, exogenous AHR ligands, and predominantly expresses AHR [8,9]. Upon ligand exposure, airway epithelial cells produce alarmins and a variety of inflammatory cytokines [10,11], accompanied by enhanced secretion of mucin and matrix metalloproteases [12]. Lung fibroblasts also switch to induce the expression of TGF-β1 and alpha-smooth muscle actin (α-SMA) [13]. These factors not only induce or exacerbate lung inflammation but also participate in airway remodeling. Although AHR activation may have deleterious effects on airway function, AHR-null mice show enhanced inflammatory responses in response to ovalbumin challenge [14,15,16]. This discrepancy may be attributed to the complexity of the AHR pathways among different cell types. AHR activation in some immune cells favors the anti-inflammatory phenotype [7], and AHR-related epigenetic regulation involving the ten-eleven translocation 1 (TET1) methylation alleviates interferon activation, protecting bronchial epithelial cells against asthma features [17,18,19].

Nuclear AHR is exported out of the nucleus and rapidly degraded by the ubiquitin-proteasome pathway to abrogate the AHR genomic pathway. This negative feedback loop prevents overactivation by agonists [6]. Thus, a reduced AHR content is frequently found in people with chronic exposure to exogenous AHR ligands and is accompanied by a series of pathologies, including asthma, chronic obstructive pulmonary disease (COPD), and lung cancer [20]. Recent studies have supported that the cytoplasmic AHR protein, either naïve or in the presence of a specific ligand, also displays unique physiological functions (the non-genomic pathway) [21]. For example, AHR has been identified as a novel adaptor of E3 ubiquitin ligase, which facilitates the assembly of the ubiquitin ligase complex and accelerates the proteolysis of certain nuclear receptors [22,23]. Src kinase [24], vimentin [25], BCL2, and adenovirus E1B 19-kDa-interacting protein 3 (BNIP3) [26] have been characterized as substrates of the AHR protein. Therefore, the level of cytoplasmic AHR can influence the amount of these proteins and the phenotypic behaviors determined by these factors. By establishing AHR knockout (AHR-KO) models, the physiological roles of AHR can be assessed; moreover, the pathologies in which AHR may be involved can be identified. Here, we found that AHR-defective lung epithelial cells increased the expression level of leucine-rich repeats and immunoglobulin-like domains 1 (LRIG1), which functions as a negative regulator of EGFR, resulting in reduced responsiveness to EGF stimulation and a delayed proliferation rate.

## 2. Results

### 2.1. AHR-KO Clones Frequently Prolonged Cell Doubling-Time, Involving a Multitude of Changes of Cell Cycle Regulators

**Figure 1:**
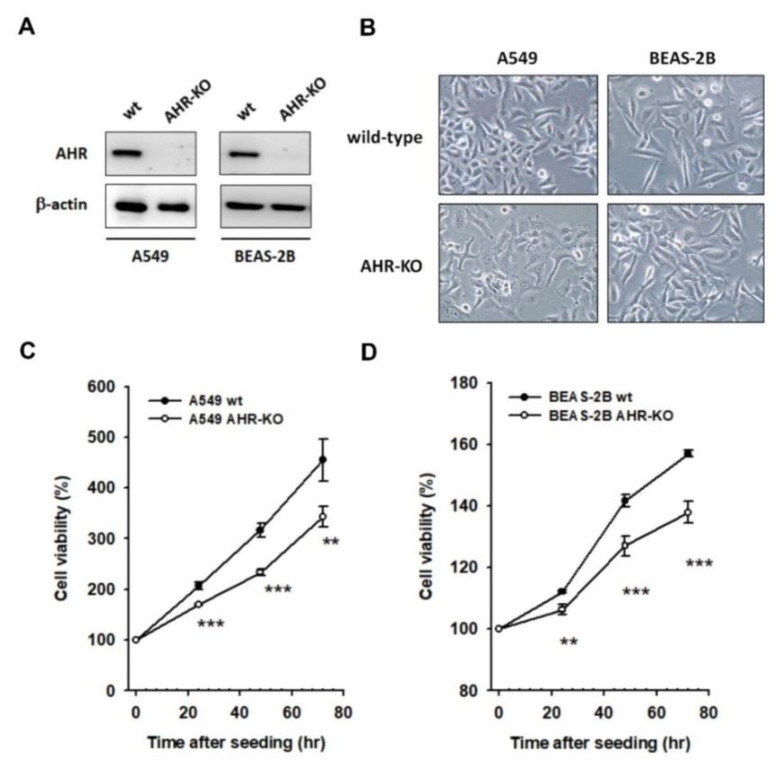
The AHR-KO clones were distinguishable to their wild-types, either in cell morphology or in cell proliferation curves. The AHR-KO cells of A549 and BEAS-2B were built by using the CRISPR/Cas9 system as described in Materials and Methods. Their expression level of AHR was affirmed by Western blotting (**A**) and cell morphology was imaged (200x) (**B**). A round cell shape with abundant nucleoli was featured in AHR-KO clones. By using MTT assay, the cell doubling time was significantly prolonged in AHR-KO clones of A549 (**C**) and BEAS-2B (**D**), compared to that of wild-type cells. (** *p* < 0.01, *** *p* < 0.001, indicates statistically significant difference from the wild-type).

The single cell-derived AHR-KO clones of A549 and BEAS-2B were built and selected as described after which the defects in AHR protein expression were authenticated (Figure 1A). The morphological features of wild-type and AHR-KO cells could be visually identified; for instance, AHR-KO cells are round and display prominent/abundant nucleoli (Figure 1B). During proliferation, AHR-KO cells frequently form small clusters with a prolonged doubling-time, as compared to their wild-types (Figure 1C,D). The morphological features and reduced proliferation potential of AHR-KO clones were also confirmed in HepG2 and ARPE-19 cell lines (Figure S1).

**Figure 2:**
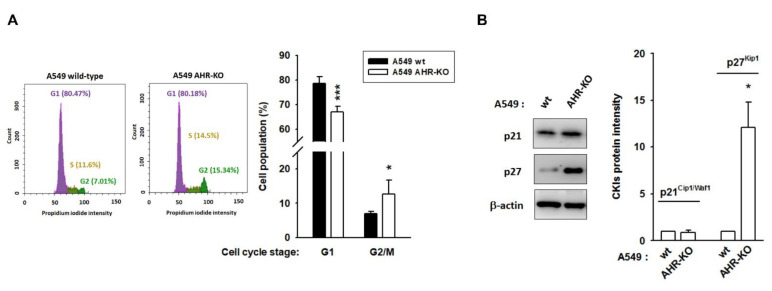
AHR-KO clones displayed G2/M phase arrest, involving the changing of a multitude of cell cycle regulators. (**A**) Using flow cytometry, the cell population of G1 phase was reduced, whereas the cell population of G2/M was increased in AHR-KO clones of A549 and BEAS-2B (Figure S2). (**B**) The expression levels of cell cycle regulators were examined. The G2/M arrest of A549 AHR-KO was strongly correlated with the induction of p27^Kip1^, a cyclin-dependent kinase inhibitor. (* *p* < 0.05, *** *p* < 0.001, indicates statistically significant difference from the wild-type). The changes of cyclin-dependent kinases (CDKs) and cyclins are shown in Figure S3.

In both A549 and BEAS-2B cell lines, AHR defects induced cell cycle arrest at the G2/M stage. The G0/G1 population of A549 wt and A549 AHR-KO was 78.69 ± 2.63% and 67.00 ± 2.24%, respectively, whereas the G2/M population was changed to 6.96 ± 0.60% and 12.72 ± 4.05%, respectively (Figure 2A and Figure S2). Next, the expression levels of cyclins, cyclin-dependent kinases (CDKs), and CDK inhibitor proteins (CKIs) were studied in A549 wt and A549 AHR-KO. We found that arresting the cell cycle of A549 AHR-KO cells was strongly associated with either the induction of p27^Kip1^ (which inhibits CDK2 activity) (Figure 2B) or a reduction in CDK6. Meanwhile, the protein levels of CDK4, p21^Cip1/Waf1^, cyclin D, and PCNA showed no significant changes between A549 wt and A549 AHR-KO cells. Interestingly, increased expression of cyclin A, cyclin B, and CDK2 was also observed in A549 AHR-KO (Figure S3).

### 2.2. The Mitogen-Mediated Signaling Was Impaired in AHR-KO Clones

**Figure 3:**
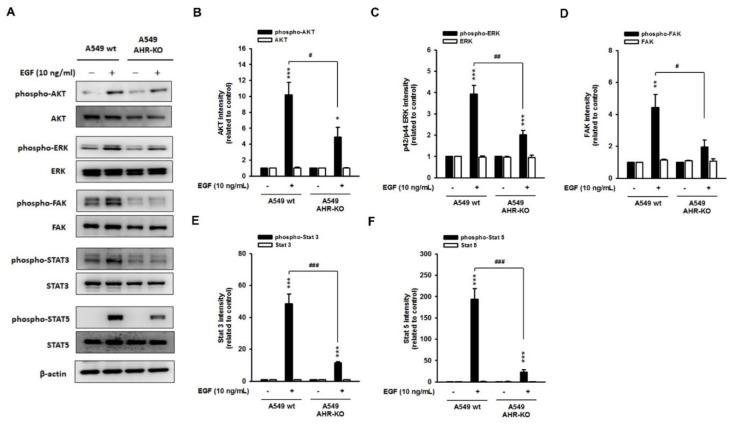
Epidermal growth factor (EGF)-mediated mitogenic pathways were alleviated in AHR-KO clones. (**A**) Representative images showed an increased phosphorylation level of AKT (protein kinase B), extracellular signal-regulated kinases (ERK), focal adhesion kinase (FAK), signal transducer, and activator of transcription (STAT) 3 and 5 upon EGF stimulation (10 ng/mL; 20 min) in A549 wt, whereas the phosphorylation status of these factors was alleviated in AHR-KO clones of A549, suggesting an impairment of EGF-induced mitogenic pathway during AHR defect. The corresponding quantitative histograms are presented in (**B**–**F**). (* *p* < 0.05, ** *p* < 0.01, *** *p* < 0.001, indicates statistically significant difference from the wild-type control; # *p* < 0.05, ## *p* < 0.01, ### *p* < 0.001, means statistical difference between the wild-type and AHR-KO after EGF stimulation). A similar response was obtained in BEAS-2B wt and AHR-KO (Figure S4).

We speculate that a non-genomic AHR pathway may be required for mitogen-mediated proliferative signaling, especially the EGF pathway. Thus, the phosphorylation level of Akt, ERK, FAK, STAT3, and STAT5 in response to EGF stimulation (10 ng/mL) was determined. We found that EGF causes rapid phosphorylation of these kinases in the wild-type cells of A549 (Figure 3) and BEAS-2B (Figure S4); however, the phosphorylation levels of EGF-induced kinases were alleviated dramatically in AHR-KO clones, as compared to those of the wild-type. The total protein level of these kinases did not change. These data strongly support the hypothesis that the prolonged cell doubling time of AHR-KO clones might be due to the attenuation of the signaling response to mitogen stimulation.

### 2.3. The Leucine-Rich Repeats and Immunoglobulin-like Domains 1 (LRIG1), an EGFR Repressor, Was Up-Regulated in AHR-KO Clones

**Figure 4:**
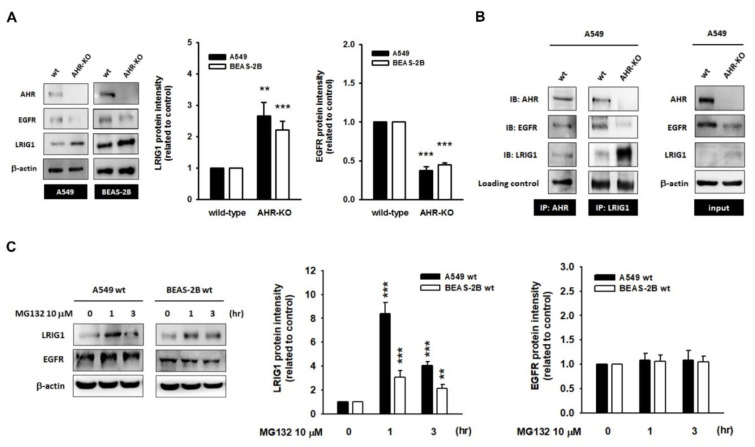
The expression of leucine-rich repeats and immunoglobulin-like domains 1 (LRIG1), the repressor of epidermal growth factor receptor (EGFR), was up-regulated in AHR-KO clones. (**A**) Representative images and quantitative data showed the up-regulation of LRIG1 protein level in AHR-KO clones. Contrarily, the EGFR is expressed in the opposite manner of LRIG1. (**B**) By using immunoprecipitation assay, the protein complex comprising of AHR, EGFR, and LRIG1 was demonstrated in A549 wt. However, in AHR-KO clones, less EGFR was associated with LRIG1. (**C**) A short-term incubation with MG132 (10 μM; proteasome inhibitor) obviously boosted LRIG1 protein level in A549 and BEAS-2B, but without alternations on EGFR. These data suggest the interaction of LRIG1 to AHR might direct LRIG1 toward proteasome-dependent degradation. (** *p* < 0.01, *** *p* < 0.001, indicates statistically significant difference from the wild-type control).

Next, we assumed that the attenuation of EGF-induced signaling in AHR-KO clones might result from the hypo-expression of EGFR, as well as the activation of negative co-receptors of EGFR. As shown in Figure 4A, we confirmed that the expression level of EGFR was reduced and that it coincided with the accumulation of the LRIG1 protein in the AHR-KO clones of A549 and BEAS-2B, as compared to that of wild-type cells. By using a co-immunoprecipitation assay, we demonstrated the protein–protein interactions among AHR, EGFR, and LRIG1 in A549 wt. It is intriguing that the absence of AHR protein obviously boosted the LRIG1 protein content in the protein complex (Figure 4B). As aforementioned, by interacting with AHR protein, AHR could facilitate the target protein’s degradation through the ubiquitin-proteasome pathway [22,23]. Treatment with proteasome inhibitor MG132 (10 μM) caused a rapid accumulation of LRIG1 in wild-types of A549 and BEAS-2B, however, it had no effects on the EGFR protein level (Figure 4C). Moreover, MG132 also improved LRIG1 levels in AHR-KO clones (Figure S5). These data substantially support the hypothesis that the cytosolic LRIG1 level is related to AHR and proteasome activity, whereas LRIG1-mediated EGFR degradation is independent of the proteasome.

### 2.4. A Disintegrin and Metalloprotease 17 (ADAM17) Activity Is Required for LRIG1-Mediated EGFR Degradation

**Figure 5:**
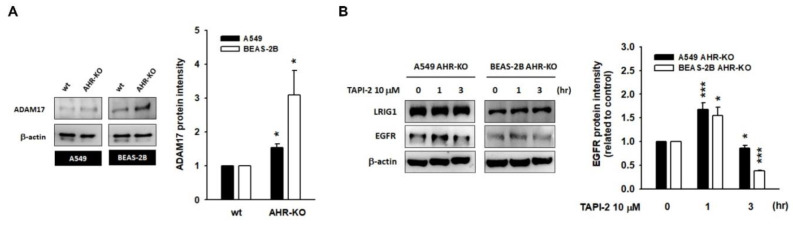
LRIG1-mediated EGFR degradation may be a disintegrin and metalloprotease 17 (ADAM17)-dependent. (**A**) Representative images and quantitative histograms showed induction of ADAM17, a membrane-bound metalloproteinase, in AHR-KO clones of A549 and BEAS-2B. (**B**) After 1 h incubation with TAPI-2 (10 μM; ADAM17 inhibitor), the EGFR level was elevated obviously; however, the EGFR returned to degradation at 3 h, either in A549 AHR-KO or in BEAS-2B AHR-KO. These data suggest LRIG1-mediated EGFR degradation is ADAM17-dependent and is a continuously active process. (* *p* < 0.05, *** *p* < 0.001, indicates statistically significant difference from the wild-type control).

It is intriguing to find the hyper-expression of ADAM17, a membrane-bound metalloprotease, in AHR-KO clones of A549 and BEAS-2B cells (Figure 5A). Short-term treatment with ADAM17 inhibitor TAPI-2 (10 μM) not only elevated EGFR protein levels in wild-type A549 and BEAS-2B cells (Figure S6) but also notably rescued the EGFR protein levels of AHR-KO clones (Figure 5B). These data proved that the AHR defect let either LRIG1 escape from proteasome degradation or ADAM17 overactivation. Both of them contribute to EGFR degradation, conferring a lower response to EGF stimulation and a prolonged cell double time.

### 2.5. The Expression of AHR and LRIG1 Was Correlated Inversely In Vivo; Furthermore, an Enhanced LRIG1 Expression Was Found in Lung Tissues of COPD Patients

**Figure 6:**
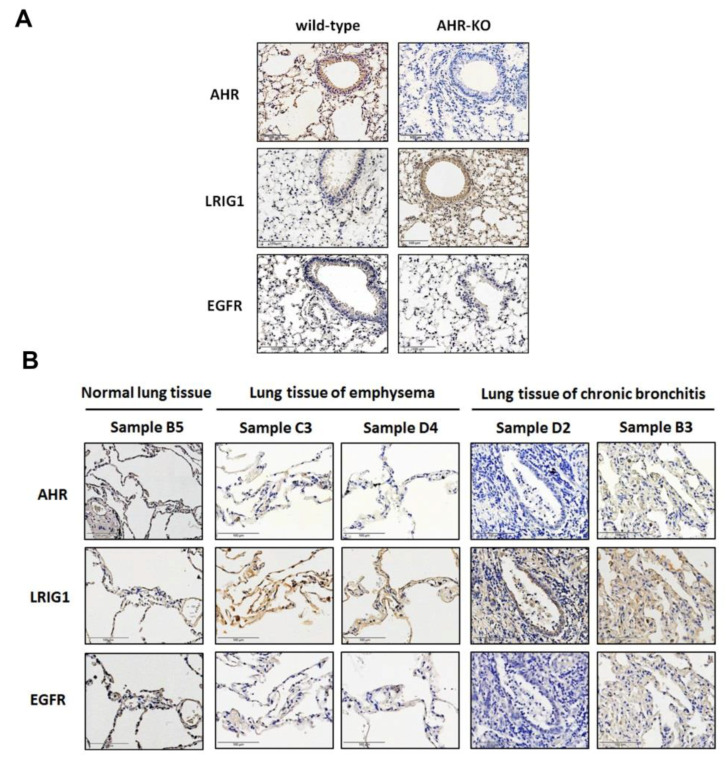
The expression of AHR, LRIG1, and EGFR in lung tissues, involving mice and humans. (**A**) AHR was undetectable in samples obtained from AHR-KO (C57BL/6−Ahr^tm1.2Arte^) mice. Representative images showed the inverse expression between AHR (or EGFR) and LRIG1 (*n* = 5). (**B**) The expression level of AHR, LRIG1, and EGFR in normal, emphysema, and chronic bronchitis lung tissues were detected. We found an apparent LRIG1 reactivity, coincided with poor AHR (or EGFR) reactivity in emphysema (*n* = 2) and chronic inflammatory lung tissues (*n* = 5). The upregulation of LRIG1 expression correlated negatively with AHR (or EGFR). The donor’s gender, age, and pathology diagnosis are summarized in Table S2. The high-magnification images are shown in Figure S7.

Finally, we confirmed the upregulation of the LRIG1 protein expression, accompanied by low expression of EGFR in the airway epithelium of AHR-KO mice. In contrast, notable reactivity of AHR and EGFR, rather than LRIG1, was observed in wild-type mice (Figure 6A). The expression correlation between AHR, LRIG1, and EGFR was examined in normal, emphysema, and chronically inflamed lung tissues (emphysema and chronic bronchitis are typical symptoms of COPD). Strong LRIG1 reactivity was detected in lung tissues of emphysema and chronic bronchitis, rather than in normal lung tissue. Contrarily, the AHR and EGFR were poorly expressed in emphysema and chronic bronchitis lung tissues but were dominantly found in epithelial cells of normal lung tissue. This data indicated a negative correlation of AHR (or EGFR) and LRIG1 expression during the pathogenesis of COPD (Figure 6B).

## 3. Discussion

Maintaining the integrity of airway epithelium is of prime importance to maintain the physical barrier function as well as allow respiration to occur at its maximum efficiency. Exposure to airborne particulates, tobacco smoke, allergens, microorganisms, and ozone are thought to induce airway inflammation and injure airway epithelium. As previously mentioned, some of these factors are AHR active and can impair airway homeostasis via the AHR genomic pathway [19,27]. Additionally, these factors might ablate the AHR content [27], which is frequently seen in aging [28] and COPD [29,30]. In this study, we confirmed that patients with emphysema and chronic lung inflammation have a significant reduction in AHR in pulmonary cells. After chronic exposure to cigarette smoke, AHR-KO mice develop airspace enlargement concomitant with a decline in lung function [30], suggesting that loss of AHR may be a susceptibility factor for COPD. The healing of airway epithelium requires complex processes involving the spreading and migration of healthy epithelial cells from neighboring sites, cell proliferation, and differentiation. An abnormal bronchial epithelial repair, paralleling with epithelial remodeling was commonly observed in COPD [31]. In a naphthalene-induced lung injury model, rapid induction of Ki67 expression was observed in airway epithelial cells. The Ki67 level plateaued until 21 days after injury in wild-type mice but returned to basal levels by 21 days in AHR-KO mice. The pattern of Ki67 dynamics revealed a delayed regeneration potential associated with AHR defects [32]. To explore the role of the AHR protein, AHR-KO clones were generated. Since primary human airway epithelial cells are difficult to transfect at high efficiency and may not survive harsh selection conditions, in this study, we successfully established AHR-KO clones from cancerous cell lines (A549 and HepG2) and immortalized normal cell lines (BEAS-2B and ARPE-19). We found several de-differentiated morphological features (including round morphology, scant cytoplasm, and prominent nucleoli), while AHR-KO, which is consistent with previous studies [32,33]. Moreover, these clones showed a reduced rate of proliferation. Taken together, the endogenous AHR protein may have growth-promoting effects, and AHR defects may prolong cell doubling time.

The growth-promoting effect of AHR is substantially strengthened by the observations that AHR is abundant in cancerous cells of numerous origins, relative to precancerous cells. Ligand treatments, such as dioxin, usually inhibit the G1 to S phase transition [34]. The G1 to S checkpoint is controlled by protein complexes comprising cyclin D, CDK4, and CDK6. In the absence of exogenous ligands, cytoplasmic AHR has been shown to interact with CDK4. Exposure to AHR agonists disrupts this interaction, reduces RB1 phosphorylation, and arrests the cell cycle in the G1 phase [35]. Early studies from AHR-silenced HepG2 similarly identified the prolonged G1 phase, involving the down-regulation of cyclin D1, cyclin E, CDK2, and CDK4 [36]; whereas in AHR knock-down keratinocytes, the growth-promoting effect of AHR was compromised by the overexpression of p27^Kip1^ and the reduction in CDK2 [37]. Embryonic fibroblasts derived from AHR-KO mice also exhibited a slower proliferation rate, concomitant with the down-regulation of G2/M kinases, CDC2, and PLK [38]. In addition, the AHR-KO fibroblasts regained proliferating potential either by the reintroduction of AHR or by overexpression of mutant E1A (adenovirus oncoprotein) [39]. In another study, deletion of AHR in lung fibroblasts either inhibited cell proliferation or increased apoptosis through the down-regulation of cellular microRNA-196a levels [40]. From our data, we found that the CDK6 protein level was reduced, whereas the p27^Kip1^ was strongly increased in AHR-KO cells. The expression levels of CDK2, cyclin A, and cyclin B were up-regulated, which might be a compensatory response. No obvious changes involving CDK4, cyclin D, PCNA, and p21^Cip1/Waf1^ were observed. Basically, these data support the growth-promoting role of AHR; however, the cell cycle regulators responding to AHR defects might be cell-specific. 

Recently, increasing evidence has suggested that endogenous AHR may directly participate in the growth-promoting signaling of keratinocyte growth factor (KGF) [41], insulin-like growth factor 2 (IGF2) [42], platelet-derived growth factor (PDGF), and basic fibroblast growth factor (bFGF) [43]. These studies reveal a potent, physiological function of AHR required for mitogenic signaling. In this study, we found that mitogen-induced transmembrane signaling was attenuated in AHR-KO clones, as compared to that in wild-type cells. After a short-term exposure to EGF, the downstream effectors of EGFR, involving the p42/p44 ERK, AKT, FAK, STAT3, and STAT5, were activated immediately; however, the amplitude was dramatically alleviated in AHR-KO clones, as compared to that in the wild-types, suggesting the disability of EGFR in the absence of AHR. Down-regulation of the EGFR level in AHR-KO clones was observed to be accompanied by an accumulation of leucine-rich repeat and immunoglobulin-like domain protein-1 (LRIG1).

LRIG1 is a type-1 transmembrane protein and is known to negatively regulate a multitude of receptor tyrosine kinase, including the ErbB family (involving the mutant EGFRvIII) [44], Ret [45], Met [46], tropomyosin-related kinase (TrkB) [47], platelet-derived growth factor receptor [48], and insulin-like growth factor receptor 1 [49]. Mice with genetic deletion of LRIG1 frequently displayed duodenal adenomas and hyperplasia in the skin, lung, and intestines [48], indicating that the loss of LRIG1 might heighten EGFR signaling and cause an overgrowth phenotype [44]. LRIG1 appears to bind directly to the EGFR through its ectodomain; subsequently, the intracellular domain might recruit and activate the E3 ubiquitin ligase Cbl, contributing to the accelerated rates of EGFR degradation [44]. However, either the mutant EGFR lacking the Cbl-binding site (Y1045F) or the EGFRvIII losing the ectodomain could still be efficiently degraded in LRIG1-overexpressing cells, suggesting that a Cbl-independent pathway might exist [44,46]. In this study, the protein complex comprised of LRIG1, EGFR, and AHR (a reported adaptor of E3 ubiquitin ligase) has been demonstrated in wild-type cells. Less LRIG1 but more EGFR was found in the protein complex of an AHR-expressing cell, whereas more LRIG but less EGFR was identified in the protein complex of an AHR-defective cell. A short incubation with MG132 (proteasome inhibitor) effectively restored the LRIG1 protein level, whereas the EGFR remained unaltered. This means that the level of LRIG1 is mainly determined by the rate of protein turnover through an AHR-mediated, proteasome-dependent manner. However, LRIG1-mediated EGFR degradation is regardless of the proteasome. It is intriguing that the membrane-bound metalloproteinase, a disintegrin and metalloprotease 17 (ADAM17), was up-regulated in AHR-KO clones. After a short bath with TAPI-2 (ADAM17 inhibitor), the increase in the EGFR level was demonstrated. Based on these data, we hypothesize that an AHR defect may allow LRIG1 to escape degradation and elicit LRIG1-dependent EGFR degradation involving ADAM17 activity, consequently dampening the response to EGF stimulation and inhibiting mitogenic signaling.

In this study, both in vitro and in vivo models successfully demonstrated the inverse correlation between AHR and LRIG1. AHR and LRIG1 have been reported to be expressed ubiquitously in all tissues; and they are dominantly expressed in lung epithelium, suggesting that the expression pattern of AHR and LRIG1 plays an important role during the pathogenesis of lung diseases. There is considerable evidence indicating the contribution of constitutively active AHR during pro-proliferative effects; moreover, the expression level of AHR is notable in tumor staging and malignancy. Partially, AHR-mediated LRIG1 degradation might be involved in tumorigenesis. For example, in malignant lung tumors, the LRIG1 promoter is usually hypermethylated, resulting in poor expression of LRIG1 and the hyperactivation of EGFR signaling [49]. This epigenetic regulation of LRIG1 was concordant with reports of colorectal cancer and cholangiocarcinoma [50,51]. On the contrary, the overexpression of LRIG1 alleviates the expression level of EGFR (either mutant or wild-type) and suppresses tumor expansion [49]. The amount of AHR protein is dynamic and is affected by several factors; for example, aging, smoking, and TGF-β1 exposure usually decrease the cytoplasmic AHR protein level [27,28]. These factors are also hazardous to the airway epithelium. Moreover, reduced AHR levels accompanied by LRIG1 accumulation were observed in COPD-derived lung tissues. By elevating the levels of LRIG1, the EGFR/PI-3 kinase/AKT, EGFR/MEK/MAPK, and EGFR/STAT signaling pathways might be mitigated, leading to hyporeactivity of the epithelium during tissue regeneration. In line with this notion, AHR-deficient lung fibroblasts also reduced the level of superoxide dismutase, resulting in increased vulnerability to oxidative stress [52]. Taken together, AHR exerts protective effects in the lungs by suppressing oxidative stress, attenuating inflammation, and reducing the loss of lung structural cells by promoting cellular proliferation and anti-apoptosis.

In this study, we first noted the up-regulation of LRIG1, the repressor of EGFR, in AHR-defective epithelial cells of lung origin. We also affirmed the lower AHR but higher LRIG1 in lung tissues of COPD. Although the overexpression of LRIG1 in cancer cells may effectively suppress the growth of the tumor, in normal tissues, the accumulation of LRIG1 might delay tissue repair and aggravate the illness. We also explored proteasome and ADAM17 activity requirements for AHR-mediated LRIG1 degradation and LRIG1-mediated EGFR degradation, respectively. These findings provide insights into the development of therapeutic strategies against AHR-related pathologies.

## 4. Materials and Methods

### 4.1. Cell Culture

Human lung epithelial cell line A549 (the non-small cell lung cancer cell) and human immortalized normal bronchial epithelial cell line BEAS-2B were purchased from Bioresource Collection and Research Center (BCRC, Hsinchu, Taiwan) and cultured in DMEM and RPMI-1640 medium, respectively, both of which were supplemented with 10% fetal bovine serum (FBS), 2 mM L-glutamine, and antibiotics. Cells were grown in 5% CO_2_ in an air-humidified incubator at 37 °C. The culture media was refreshed every 2 days.

### 4.2. Plasmids, Transfection and Generating AHR Knockout Clones with CRISPR/Cas9 System

The plasmids (CRISPR/Cas9 knockout plasmid and homology-directed repair plasmid) were obtained from Santa Cruz Biotechnology, Inc. (Dallas, TX, USA). Purified plasmids were co-transfected into A549 (or BEAS-2B) cells using Trubofect^TM^ reagent (Thermo Scientific, Waltham, MA, USA). After a 4-week selection period, AHR-KO colonies were isolated by their resistance to puromycin (1 μg/mL), followed by Western blotting to affirm the homozygous deletions of AHR. To avoid off-target effects, the N value throughout the study represents the number of testing clones.

### 4.3. Cell Proliferation Curve Determination (MTT Assay)

Cells were inoculated in a 48-well culture dish at a density of 1 × 10^4^ cells/well, and after 24, 48, and 72 h, the cell activity of succinate dehydrogenase was quantified by the conversion of MTT [3-(4,5-dimethyl-2-thiazolyl)-2,5-diphenyl-2H-tetrazolium bromide] to formazan. Briefly, cells were incubated with culture medium in combination with MTT (100 μg/mL) for 2 h. Then, the supernatant was discarded and the DMSO was added to elute the formazan crystals. We then measured the absorbance of the samples at 570 nm against the control group (day 0), using a multiwall plate reader (Chromate 4300, Awareness Technology, Inc., Palm City, FL, USA). Cell proliferation rate (%) = (A_experimental group_ − A_control_)/A_control_ × 100%, where A_control_ represents the A_570_ value at the beginning of the study.

### 4.4. Flow Cytometric Analysis of Cell Cycle

The cells were grown in a 60 mm cell culture dish and were harvested at 70% confluency. Briefly, cells were trypsinized, washed with PBS twice, and followed by a 4 °C overnight fixation with 70% ethanol. Afterwards, the cells were incubated with propidium iodide (50 μg/mL) and RNase A (10 μg/mL) at 37 °C for 30 min and then analyzed by CytoFLEXTM flow cytometry (Beckman Coulter, Inc., Brea, CA, USA).

### 4.5. Protein Extraction and Western Blotting

Cells were lysed by an adequate volume of RIPA lysis buffer. The whole-cell lysate was centrifuged at 14,000× *g* for 15 min to remove cell debris, and the protein in the supernatant was quantified using Bradford reagent (Bio-Rad Laboratories Inc., Hercules, CA, USA). Samples were mixed with 4× Laemmli sample buffer, and equal amounts (30 μg/well) of protein were separated by sodium dodecyl sulfonate-polyacrylamide gel electrophoresis (SDS-PAGE), followed by electrophoretic transfer to polyvinylidene fluoride (PVDF) membrane. Next, membrane blocking was performed by incubation with 5% skimmed milk, subsequently, the membrane was reacted to primary antibodies (Table S1) overnight at 4 °C. After routine washing, the blots were incubated with secondary antibodies (horseradish peroxidase-conjugated) and the enhanced chemiluminescence (ECL) reagent (Millipore, Billerica, MA, USA) was added for protein signal development. Finally, the density of the protein signal was automatically quantified using the Gel-Pro analyzer software (version 4.0, Media Cybernetics, Rockville, MD, USA) [53].

### 4.6. Immunoprecipitation

Cell lysates were prepared as described earlier. A small amount of protein A magnetic beads (Millipore) was added to pre-clear nonspecific binding. Next, with the combination of captured antibodies (1 μg/mL), the pre-cleared lysates (0.5 mg) were gently inverted overnight at 4 °C in a refrigerator. The antibodies used in this study were antibodies against AHR (Santa Crus, CA, USA, catalog no. sc-74571) and LRIG1 (Genetex, catalog no. GTX119485). The next day, 100 μL of protein A magnetic beads were added to pull-down the immunocomplex. After a routine washing, the protein immunocomplex was denatured using 2× Laemmli sample buffer (100 µL/reaction) with 95 °C heating for 5 min. Samples were separated on a 10% SDS-PAGE and analyzed as described previously.

### 4.7. Immunohistochemistry

The lung specimens of wild-type and AHR-KO mice were a gift from Dr. Lee CC [54]. The human lung disease and normal tissue array were obtained from US Biomax, Inc., Derwood, MD, USA (catalog no. LUD481). The expression patterns of AHR, LRIG1, and EGFR were detected by using specific primary antibodies (anti-AHR, Santa Cruz, catalog no. sc-74571; anti-LRIG1 and anti-EGFR, Genetex, catalog no. GTX119485 and GTX121919, respectively) according to the standard protocol of Bio-Check Laboratories Ltd. (New Taipei City, Taiwan). Peroxidase-labeled specimens were observed on a Nikon light microscope equipped with a Polychrome-III camera (YC technology, New Taipei City, Taiwan) and Image Eye software (FMJ Software, Stockholm, Sweden).

### 4.8. Statistical Analysis

Each experiment included at least three independent studies. All quantitative data were expressed as the mean ± standard error of the mean (SEM). Group differences were compared with Student’s *t*-test using SPSS software (version 18.0, SPSS Inc., Chicago, IL, USA). *p* < 0.05 was considered as statistically significant.

## Data Availability

Data presented in this study are available on request from the corresponding author.

## Supplementary Materials

The following are available online at https://www.mdpi.com/article/10.3390/ijms22189988/s1.

## Abbreviations

EGF	Epidermal growth factor
EGFR	Epidermal growth factor receptor
MAPK	Mitogen-activated protein kinase
Akt	Protein kinase B
STAT	Signal transducer and activator of transcription
AHR	Aryl hydrocarbon receptor
LRIG1	Leucine-rich repeats and immunoglobulin-like domains 1
COPD	Chronic obstructive pulmonary disease
KO	Knock-out
ADAM17	A disintegrin and metalloprotease 17
CDK	Cyclin-dependent kinase
