# Supplementary material for: Aryl Hydrocarbon Receptor Defect Attenuates Mitogen-Activated Signaling through Leucine-Rich Repeats and Immunoglobulin-like Domains 1 (LRIG1)-Dependent EGFR Degradation
Source: Int J Mol Sci. 2021 Sep 15;22(18):9988. doi: 10.3390/ijms22189988 (PMC8464816; doi:10.3390/ijms22189988)
Supplement: Supplementary file 1 [file ijms-22-09988-s001.zip › ijms-1329256-supplementary.pdf]

International Journal of *Molecular Sciences*

MDPI

Supplementary Materials

Table S1. Antibodies for Western blotting

Antigen Target	Catalog No.	Source
AHR	sc-74571	Santa Cruz
AHR	GTX22770	GeneTex
p27	sc-1641	Santa Cruz
ERK	sc-93	Santa Cruz
ERK (phospho Y204)	sc-7383	Santa Cruz
Akt	sc-8312	Santa Cruz
Akt (phospho S473)	9271S	Cell Signaling
FAK	ab40794	Abcam
FAK (phospho Y397)	CSB-PA040073	CUSABIO
STAT3	9139S	Cell Signaling
STAT3 (phospho Y705)	ab76315	Abcam
STAT5	94205S	Cell Signaling
STAT5 (phospho Y694)	9359S	Cell Signaling
EGFR	GTX121919	GeneTex
LRIG1	GTX119485	GeneTex
LRIG1	bs1844R	BIOSS
ADAM17	ab2051	Abcam
β-actin	A5441	Sigma-Aldrich

Table S2. Basic information of donors whose tissues were analyzed using the tissue array

Tissue ID	Gender	Age	Pathology Diagnosis	Anatomic site
A1, B1	M	35	Trachea (from autopsy)	Trachea
A5, B5*	M	19	Normal lung	Lung
A7, B7	M	48	Normal lung	Lung
A3, B3*	M	51	Chronic bronchitis (small bronchus)	Lung
A8, B8	F	57	Chronic bronchitis with goblet cell hyperplasia	Lung
C1, D1	M	61	Chronic bronchitis	Lung
C2, D2*	M	65	Bronchiectasis, inflammatory	Lung
C6, D6	M	56	Tuberculosis, inflammatory	Lung
C3*, D3	M	66	Emphysema	Lung
C4, D4*	F	50	Emphysema	Lung

Note: * was used as a representative image.

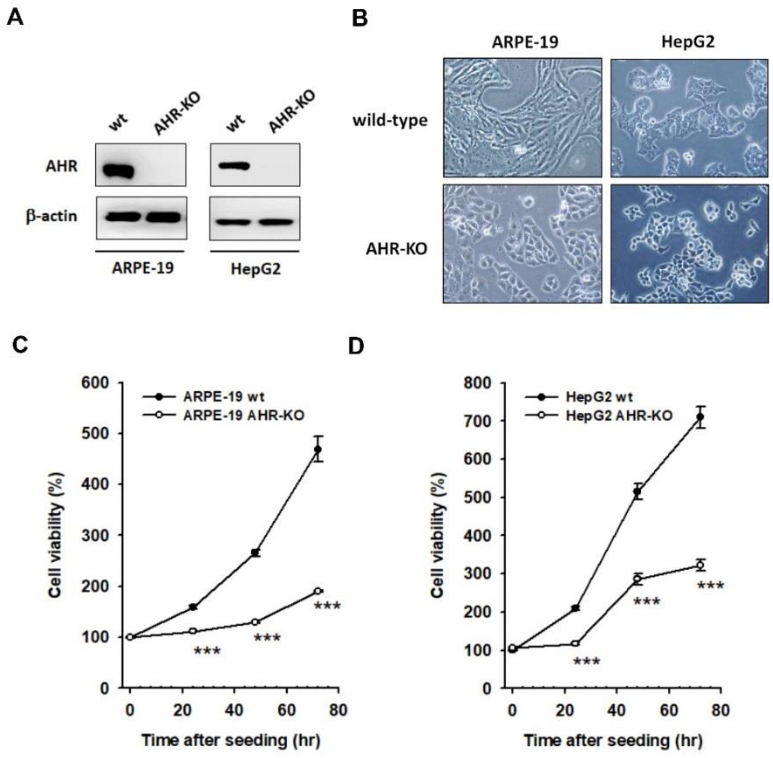

Figure S1. The cell morphology and cell proliferation curve of AHR-KO clones. The AHR-KO cells derived from ARPE-19 (normal human retinal pigmented epithelium) and HepG2 (hepatocellular carcinoma) cells were generated using CRISPR/Cas9 system as described in Materials & Methods. The expression of AHR was affirmed by western blotting (A) and cell morphology was confirmed by performing imaging (B). A de-differentiated morphology was observed in AHR-KO clones. (C) MTT assay revealed that the cell doubling time was significantly prolonged in AHR-KO clones. (***$p <$ 0.001, indicates statistically significant difference compared to the wild-type).

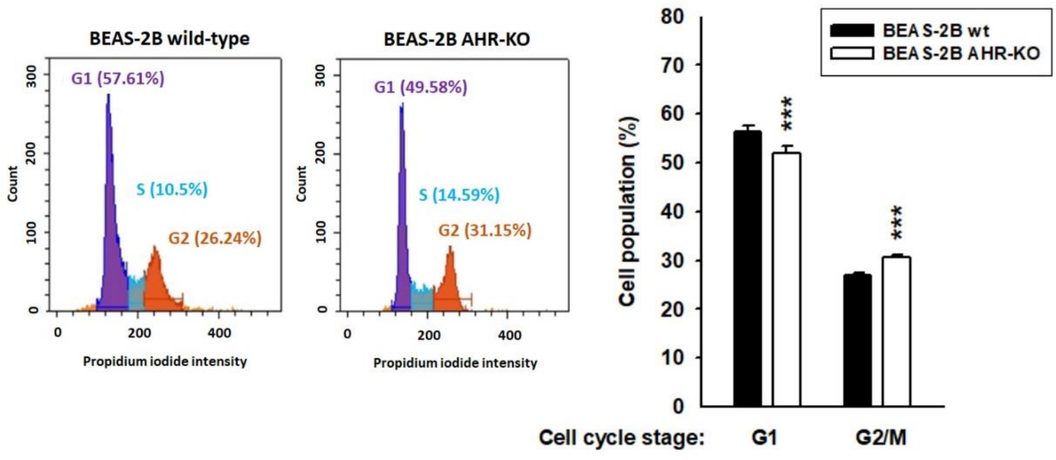

Figure S2. The AHR-KO clones of BEAS-2B displayed cell cycle arrest at G2/M phase. The cell population of G0/G1, S (synthesis), and G2/M was determined by propidium iodide staining and flow cytometry. The G0/G1 population of BEAS-2B wt and AHR-KO was 56.42 ± 1.08% and 51.94 ± 1.38%; whereas the G2/M population was changed to 26.82 ± 0.48% and 30.49 ± 0.50%, respectively. (***p < 0.001, indicates statistically significant difference from the wild-type).

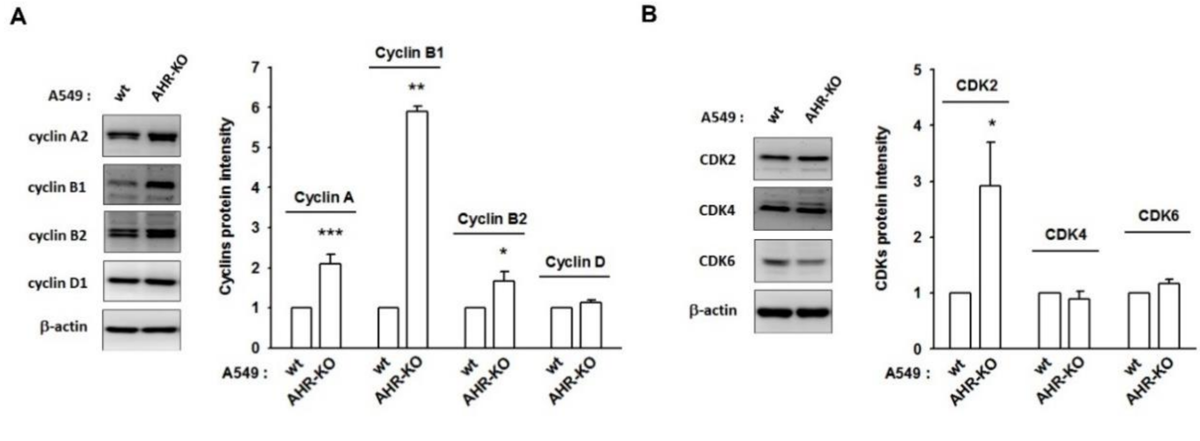

Figure S3. A multitude of cell cycle regulators was changed in A549 AHR-KO. The whole lysate was prepared, and the level of cyclins (A) and cyclin-dependent kinases (CDKs) (B) was evaluated and quantified. Data showed the up-regulation of cyclin A, cyclin B1/B2, and CDK2 in A549 AHR-KO compared to those of A549 wt. These might be compensatory responses. The level of cyclin D, CDK4, and CDK6 remained unaltered. (*$p < 0.01$, **$p < 0.05$, ***$p < 0.001$, indicates statistically significant difference from the wild-type).

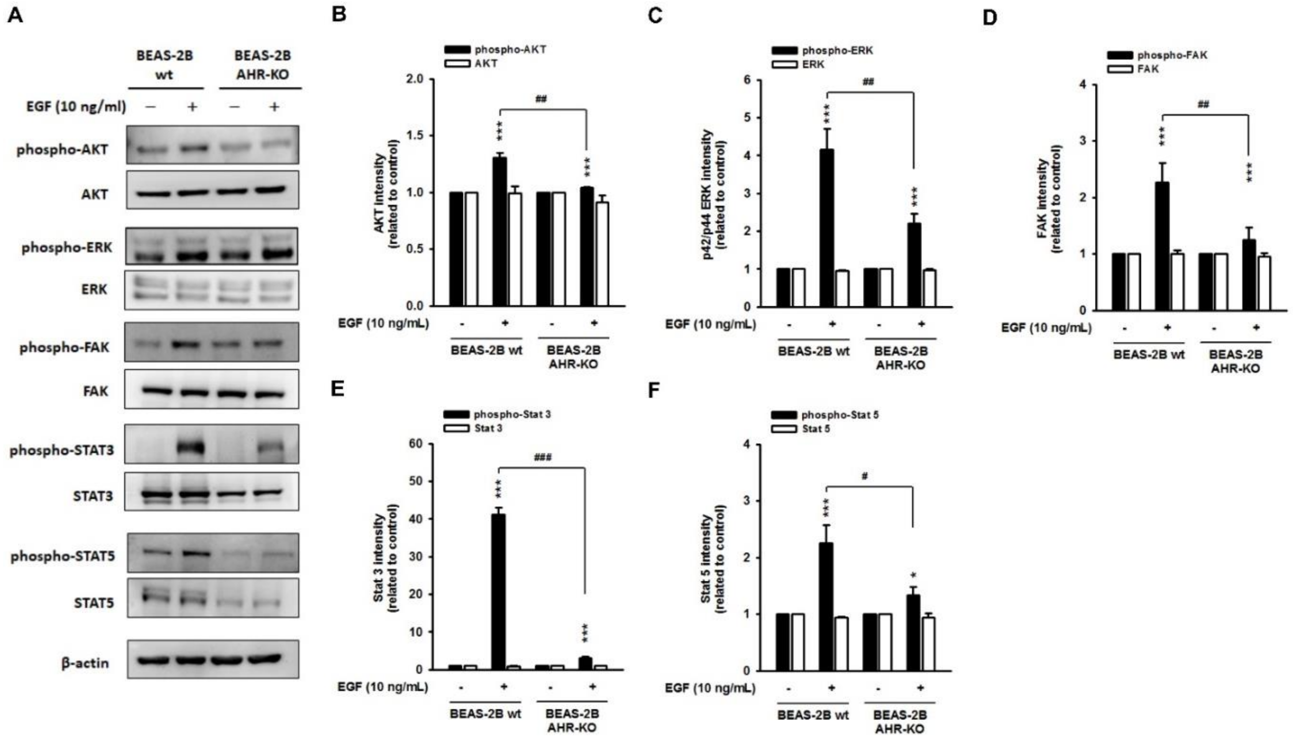

Figure S4. Epidermal growth factor (EGF)-mediated mitogenic pathways were alleviated in BEAS-2B AHR-KO. (A) Representative images showed an increased phosphorylation level of AKT (protein kinase B), extracellular signal-regulated kinases (ERK), focal adhesion kinase (FAK), signal transducer and activator of transcription (STAT) 3 and 5 upon EGF stimulation (10 ng/mL; 20 min) in BEAS-2B wt, whereas the phosphorylation status of these factors was alleviated in AHR-KO clones of BEAS-2B, suggesting an impairment of EGF-induced mitogenic pathway during AHR defect. The corresponding quantitative histograms were presented in (B) to (F). (*$p < 0.05$, ***$p < 0.001$, indicates statistically significant difference from the wild-type control; #$p < 0.05$, ##$p < 0.01$, ###$p < 0.001$, means statistical difference between the wild-type and AHR-KO after EGF stimulation).

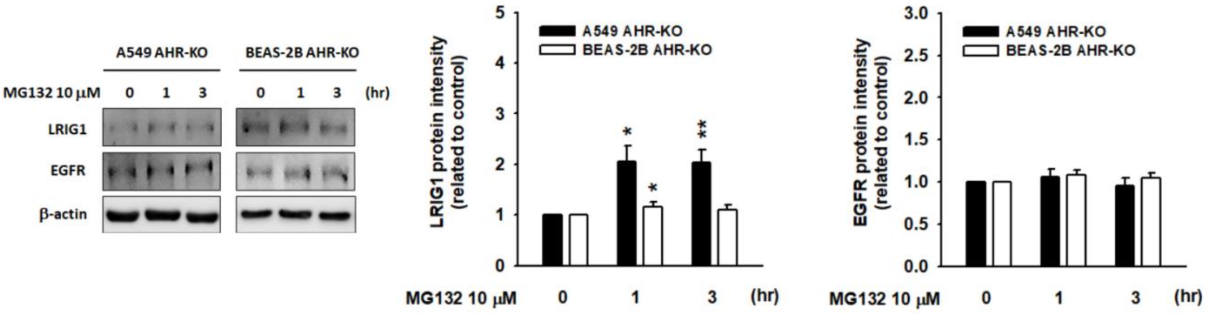

Figure S5. Proteasome inhibition prevented LRIG1 degradation in AHR-KO clones. After 1~3 hours MG132 (proteasome inhibitor) treatment, a mild up-regulation of LRIG1 protein level was found in AHR-KO clones of A549 and BEAS-2B. The expression of EGFR was not affected by MG132 treatment. This data demonstrated the crucial role of proteasome activity during LRIG1 degradation. (*$p < 0.05$, **$p < 0.01$, indicates statistically significant difference from the wild-type control).

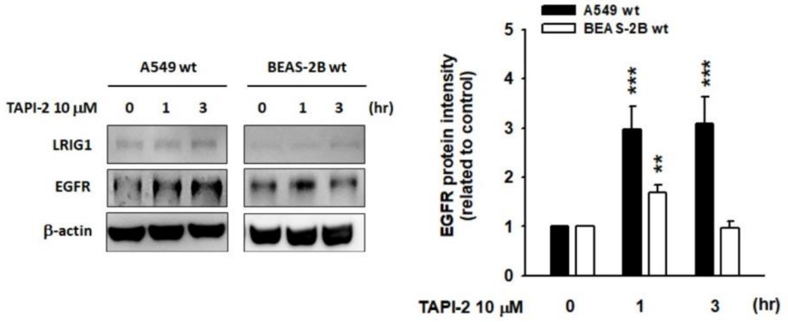

Figure S6. Disintegrin and metalloprotease 17 (ADAM17) activity is required for LRIG1-mediated EGFR degradation. After 1~3 hours TAPI-2 treatment (10 μM; ADAM17 inhibitor), the accumulation of LRIG1 was found in A549 wt and BEAS-2B wt, suggesting the requirement of ADAM17 activity during LRIG1-mediated EGFR degradation. (**$p < 0.01$, ***$p < 0.001$, indicates statistically significant difference from the wild-type control).

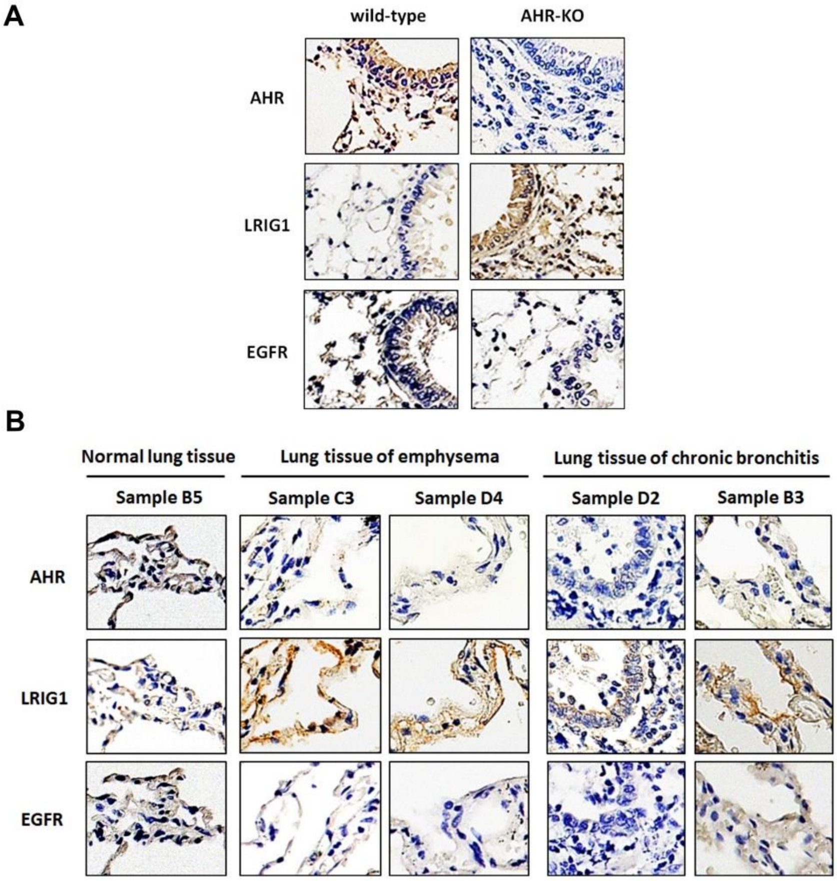

Figure S7. The expression of AHR, LRIG1 and EGFR in lung tissues, involving mice and human. (A) AHR was undetectable in samples obtained from AHR-KO (C57BL/6-Ahr$^{tm1.2Arte}$) mice. Representative images showed the inverse expression between AHR (or EGFR) and LRIG1 (n = 5). (B) The expression level of AHR, LRIG1, and EGFR in normal, emphysema, and chronic bronchitis lung tissues were detected. We found an apparent LRIG1 reactivity, coincided with poor AHR (or EGFR) reactivity in emphysema (n = 2) and chronic inflammatory lung tissues (n = 5). The upregulation of LRIG1 expression correlated negatively with AHR (or EGFR). The donor's gender, age, and pathology diagnosis are summarized in Table S2.
